# Cross‐Modal Graph Contrastive Learning with Cellular Images

**DOI:** 10.1002/advs.202404845

**Published:** 2024-06-21

**Authors:** Shuangjia Zheng, Jiahua Rao, Jixian Zhang, Lianyu Zhou, Jiancong Xie, Ethan Cohen, Wei Lu, Chengtao Li, Yuedong Yang

**Affiliations:** ^1^ Global Institute of Future Technology Shanghai Jiaotong University University Shanghai 200240 China; ^2^ School of Computer Science and Engineering Sun Yat‐sen University Guangzhou 510000 China; ^3^ Galixir Technologies Shanghai 200100 China; ^4^ School of Informatics Xiamen University Xiamen 361005 China; ^5^ IBENS, Ecole Normale Supérieure PSL Research Institute Paris France

**Keywords:** cellular image, cross‐modal learning, drug discovery, graph neural networks, self‐supervised learning

## Abstract

Constructing discriminative representations of molecules lies at the core of a number of domains such as drug discovery, chemistry, and medicine. State‐of‐the‐art methods employ graph neural networks and self‐supervised learning (SSL) to learn unlabeled data for structural representations, which can then be fine‐tuned for downstream tasks. Albeit powerful, these methods are pre‐trained solely on molecular structures and thus often struggle with tasks involved in intricate biological processes. Here, it is proposed to assist the learning of molecular representation by using the perturbed high‐content cell microscopy images at the phenotypic level. To incorporate the cross‐modal pre‐training, a unified framework is constructed to align them through multiple types of contrastive loss functions, which is proven effective in the formulated novel tasks to retrieve the molecules and corresponding images mutually. More importantly, the model can infer functional molecules according to cellular images generated by genetic perturbations. In parallel, the proposed model can transfer non‐trivially to molecular property predictions, and has shown great improvement over clinical outcome predictions. These results suggest that such cross‐modality learning can bridge molecules and phenotype to play important roles in drug discovery.

## Graphical abstract

This study introduces a novel approach to enhance molecular representation learning by integrating high‐content cell microscopy images at the phenotypic level. The proposed unified framework employs contrastive loss functions for cross‐modal pre‐training, enabling mutual retrieval of molecules and images. The model improves not only molecular properties predictions but also clinical outcome predictions, highlighting the potential of cross‐modality learning in bridging molecular structures to phenotypes for drug discovery.

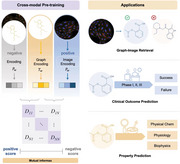

## 1 Introduction

Learning discriminative representations of molecules is a fundamental task for numerous applications such as molecular property prediction, de novo drug design, and material discovery.^[^ ^1^ ^]^ Since molecular structures are essentially topological graphs with atoms and covalent bonds, graph representation learning can be naturally introduced to capture the representation of molecules.^[^ ^2^, ^3^, ^4^, ^5^ ^]^ Despite the fruitful progress, graph neural networks (GNNs) are known to be data‐hungry, i.e., requiring a large amount of labeled data for training. However, task‐specific labeled data are far from sufficient, as wet lab experiments are resource‐intensive and time‐consuming. As a result, training datasets in chemistry and drug discovery are typically limited in size, and neural networks tend to overfit them, leading to poor generalization capability of the learned representations.

Inspired by the fruitful achievements of self‐supervision learning in computer vision^[^ ^6^, ^7^ ^]^ and natural language processing,^[^ ^8^, ^9^ ^]^ there have been a few attempts to extend self‐supervised schemes to molecular representation learning, leading to remarkable results.^[^ ^10^, ^11^, ^12^, ^13^, ^14^, ^15^ ^]^ The core of self‐supervised learning lies in establishing meaningful pre‐training objectives to harness the power of large unlabeled data. The pre‐trained neural networks can then be used to fine‐tune for small‐scale downstream tasks.

However, pre‐training on molecular graph structures remains a stiff challenge. One of the limitations of current approaches is the lack of domain knowledge in chemistry or chemical synthesis. Recent studies have pointed out that pre‐trained GNNs with random node/edge masking give limited improvements and often lead to negative transfer on downstream tasks,^[^ ^10^, ^16^, ^17^ ^]^ as the perturbations actions on graph structures can hurt the structural inductive bias of molecules. Furthermore, molecular modeling tasks often require predicting the binding/interactions between molecules and other biological entities (e.g., RNA, proteins, pathways), and further generalizing to the phenotypic/clinical outcomes caused by these specific bindings.^[^ ^18^ ^]^ Self‐supervised learning methods that solely manipulate molecular structures struggle to handle downstream tasks involving complex biological processes and thus have limited practicality in a wide range of drug discovery applications. One possible way to overcome this challenge is to assist in learning molecular representation with another modality related with molecules.

**Figure 1:**
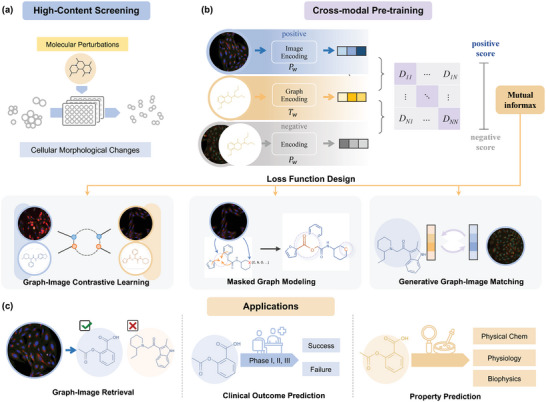
Overview of the methodology and application scenarios of MIGA. a) Illustration of high‐content screening experimental protocol. b) Pre‐training scheme of our method. Molecular graph and cellular image representations are jointly learned from pairwise data in a contrastive manner. We used three complementary contrastive objectives to perform cross‐modal pre‐training. c) Downstream applications. The learned representations and encoders are generalizable and applicable to multiple downstream tasks.

With the rapid development of biotechniques, high‐content cell microscopy imaging (HCI) is an increasingly important biotechnology in recent years in the domain of drug discovery and system biology.^[^ ^19^, ^20^, ^21^ ^]^ As shown in **Figure** **1**a, small molecules enter into cells and affect their biological functions and pathways, leading to morphological changes in cell shape, number, structure, etc., that are visible in microscopy images after staining. Analysis and modeling based on these high‐content images have shown great success in molecular bioactivity prediction,^[^ ^22^ ^]^ mechanism identification,^[^ ^23^, ^24^ ^]^ polypharmacology prediction,^[^ ^25^ ^]^ etc. The stained cell images contain rich morphological information that reflects the biological changes induced by chemical structures in cell cultures. They primarily provide a macro‐level view of cellular responses but do not directly reveal molecular structural information. Thus, we hypothesize that this phenotypic modality has complementary strengths with molecular structures to make enhanced representations and thus benefit the downstream tasks involved in intricate biological processes.

However, building connections between molecular structures and high‐content cellular images is a challenging task that highlights representative, unsolved problems in cross‐modal learning. The first challenge comes from the unclear fine‐grained correspondence between molecules and images. Unlike conventional cross‐modal paired data such as caption and picture, the patterns of the molecule are not directly reflected in the cellular image, thus preventing us from using traditional cross‐modal encoders for alignment. The second challenge arises from the noise and batch effect of the cellular data.^[^ ^26^ ^]^ For example, cellular images obtained from the same molecule can vary considerably. Existing cross‐modal pre‐training objectives may overfit to noisy images, thereby reducing the model's generalization capability.

Herein, we propose Molecular graph and hIgh content imaGe Alignment (MIGA), a novel cross‐modal graph‐and‐image pre‐training framework to address the above issues. Given the increasing abundance of high‐content cellular data, our work comprehensively utilizes high‐content cell microscopy images to assist in learning molecular representation, which is a major innovation in the field of scientific applications. We first encode the molecule and cell microscopy images independently with a molecular graph encoder and an image encoder. The encoded graph and image embeddings are then aligned through three contrastive modules: graph‐image contrastive (GIC) learning, masked graph modeling (MGM), and generative graph‐image matching (GGIM). Specifically, 1) GIC encourages high similarity between the latent embeddings of matched graph‐image pairs while pushing those of non‐matched pairs apart; 2) MGM, a local cross‐modal module that utilizes both the observed (unmasked) graph and the cell image to predict the masked molecular patterns and 3) GGIM aims to further distinguish the hard negative graph‐image pairs that share similar global semantics but differ in fine‐grained details.

Enabled by the massive publicly available high‐content screening data,^[^ ^27^ ^]^, we establish a novel cross‐modal benchmark dataset that contains 750k molecular graph‐cellular image pairs. To this end, we propose a novel cross‐modal training framework (MIGA) with multiple adaptive loss functions to comprehensively utilize high‐content cell microscopy images to assist in learning molecular representation. To evaluate models on this benchmark, we propose a new biological meaningful retrieval task specific to graph‐image cross‐modal learning. We also include existing clinical outcome prediction and property prediction tasks to further assess the learned representations. Extensive experiments demonstrate that the cross‐modal representations learned by our proposed model, MIGA, can benefit a wide range of downstream tasks that require extensive biological priors.

## 2 Results

### 2.1 MIGA Framework

At the core of our method is the idea of infusing structural representations with biological perception by building connections between molecular graph and their induced morphological features.

To achieve this, as illustrated in Figure 1b, given pairs of graph and image, we employ two independent encoders (a GNN *f* ^*g*^ and a CNN (convolutional neural networks) *f* ^*i*^) to produce the representations of a molecular graph *G* and a cellular image *I*, and align them with inter‐ and intra‐contrastive losses. This cross‐modal framework pulls the matched graph‐image pairs together and contrasts the unmatched pairs apart. After pre‐training, we use the output representations to make zero‐shot predictions or fine‐tune the networks on downstream tasks. This cross‐modal learning process can also be interpreted as morphological information being passed through the convolutional neural network to the graph neural network in a knowledge‐distillation manner.^[^ ^28^ ^]^ The framework is detailed in the Experimental Section.

### 2.2 Cross‐Modal Pre‐Training Enables Graph‐Image Mutual Retrieval

For the cross‐modal pre‐training model, the most intuitive application scenario is mutual retrieval. Here, we formulate two tasks: 1) molecular graph as query and cellular image as targets (image retrieval); 2) image as query and graph as targets (graph retrieval). Both tasks have wide applications in phenotype‐based drug discovery, especially for virtual screening and scaffold hopping. The goal of this task is to demonstrate whether the learned embeddings are able to preserve the inherent relations among corresponding graph‐image pairs. As shown in **Table** **1**, our method MIGA significantly outperforms baseline methods including ECFP+CellProfiler,^[^ ^29^ ^]^ RACR,^[^ ^30^ ^]^ UCCH,^[^ ^31^ ^]^ GraphCL,^[^ ^12^ ^]^ ALIGN,^[^ ^32^ ^]^ and CLIP.^[^ ^33^ ^]^

**Table 1:** Graph‐image retrieval tasks on a held‐out set of CIL‐750K. MIGA and its variants are compared with modified pre‐training methods, GraphCL,^[^ ^12^ ^]^ CLIP,^[^ ^33^ ^]^ ALIGN,^[^ ^32^ ^]^ along with random initialization and a feature‐based method ECFP^[^ ^29^ ^]^ + CellProfiler.^[^ ^34^ ^]^ The average of mean reciprocal rank (MRR), area under the receiver operating characteristic curve (AUC), Hit@1, Hit@5, and Hit@10 are reported. The best and second best results are marked **bold** and **bold**, respectively.

Task	Image Retrieval					Graph Retrieval				
Metrics	MRR	AUC	Hit@1	Hit@5	Hit@10	MRR	AUC	Hit@1	Hit@5	Hit@10
Random Init	0.051	0.500	0.010	0.050	0.100	0.051	0.500	0.010	0.050	0.100
ECFP+CellProfiler	0.052	0.511	0.010	0.049	0.100	0.053	0.500	0.011	0.050	0.100
RACR	0.065	0.522	0.021	0.065	0.116	0.122	0.546	0.080	0.119	0.242
UCCH	0.193	0.771	0.145	0.194	0.330	0.267	0.784	0.197	0.318	0.382
GraphCL	0.247	0.818	0.106	0.391	0.558	0.279	0.835	0.132	0.433	0.616
ALIGN	0.288	0.821	0.148	0.434	0.594	0.379	0.810	0.214	0.581	0.713
CLIP	0.265	0.890	0.105	0.437	0.635	0.327	0.878	0.171	0.515	0.672
GIC	0.288	0.847	0.148	0.434	0.594	0.280	0.907	0.125	0.447	0.652
GIC+GIM	0.303	0.876	0.145	0.485	0.660	0.337	0.927	0.176	0.523	0.693
GIC+MGM	**0.409**	0.913	**0.244**	0.612	0.741	0.405	0.931	0.244	0.598	0.737
GIC+MGM+GIM	0.401	**0.926**	0.230	**0.616**	**0.743**	**0.433**	**0.935**	**0.275**	**0.628**	**0.739**
MIGA (Full)	**0.417**	**0.936**	**0.248**	**0.623**	**0.748**	**0.413**	**0.940**	**0.249**	**0.614**	**0.739**

To be specific, MIGA achieves 15.2% absolute MRR gain, 18.6% absolute Hit@5 gain (t‐test: *p*‐value = 1.95e‐48), and 11.3% absolute Hit@10 gain (t‐test: *p*‐value = 3.37e‐23) over the best baseline CLIP on the image retrieval task. When comparing with the straightforward applications (RACR and UCCH), those results highlight the necessity and effectiveness of our novel approach to establish connections between molecular structures and high‐content cellular images. These improvements are also consistent in the graph retrieval tasks. Three contrastive learning modules are also validated by ablation studies. As shown in Section F (Supporting Information), relative to the basic loss (GIC), adding MGM and GIM both substantially improves the pre‐trained model's performance across two tasks. This is in line with our expectation as GIC operates on unimodal representations while MGM and GIM operate on cross‐modal representations. The proposed GGIM further enhances the model by reducing the noise and building explicit connections between the two modalities. Further ablation studies in different weights of contrastive learning modules, architecture choices, and usage of cellular images demonstrated that the current model architecture and training scheme were optimal for our prediction task (Section F, Supporting Information).

**Figure 2:**
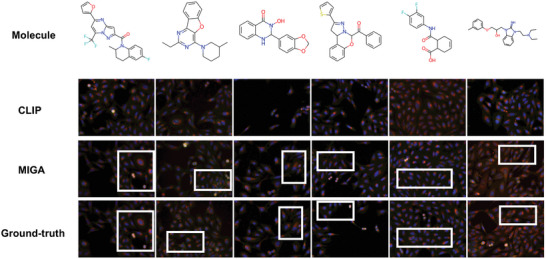
Examples of image retrieval tasks. The query molecules and the top‐ranked images being retrieved from different methods and the ground‐truth images are shown. The main differences between CLIP, MIGA, and Ground‐truth are highlighted in the box.

To indicate our results, we randomly picked six molecules and the retrieved best images by CLIP and our model (**Figure** **2**). Obviously, our model retrieved more similar images to the ground‐truth ones than CLIP (more showcases are included in the Sections C and D, Supporting Information).

### 2.3 Investigation of MIGA Representation

**Figure 3:**
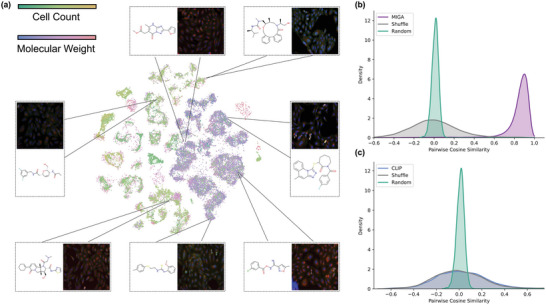
Investigation of MIGA representation. a) The representations used in this study were extracted from a held‐out set of the pre‐training dataset, which included 3000 unique molecules and their corresponding images. The two‐color scales, representing cell count and molecular weight, are used to distinguish the two different modalities of the same molecule. Each point in the resulting plot is colored according to its corresponding molecular weight (cellular image) or cell count (molecular graph). The plot also highlights several closely paired samples within the latent space. b) Distribution of molecular and image pairwise cosine similarity of MIGA and baselines. c) Distribution of molecular and image pairwise cosine similarity of CLIP and baselines.

To visually examine the representations learned by self‐supervised tasks, we utilized the t‐SNE algorithm^[^ ^35^ ^]^ to map two modalities in a unified 2D space. As depicted in **Figure** **3**a, we present the representation of 3000 molecules and their corresponding images from the held‐out set, embedded into 2D using t‐SNE, and colored based on molecular weights (cellular image embeddings) and cell count (molecular graph embedding), respectively. MIGA effectively learns close representations for paired molecules and images, indicating that the proposed three contrastive modules can learn a common low‐dimensional space to embed biologically meaningful graphs and images. This is also supported by our Figure 3b,c. MIGA achieves significantly higher similarity between the learned molecule and image embeddings for the original pairs than the shuffled (Figure 3b). In contrast, the CLIP method, which emphasizes homogeneity, produces molecules and images that are not well‐matched and remain the same before and after the shuffle (Figure 3c). These results demonstrate that our model can effectively correspond to the distributions from the two modalities, and thus could achieve performance gains on multiple downstream tasks.

### 2.4 MIGA Retrieves Functional Molecules From Unseen Genetic Perturbations

**Figure 4:**
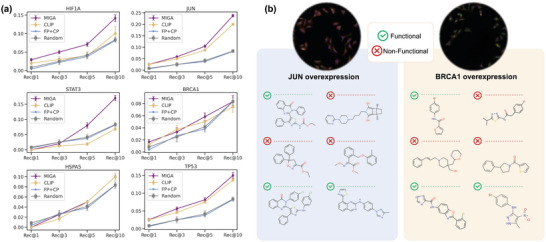
Retrieving functional molecules from unseen genetic perturbations. a) Performance of different methods to retrieve functional compounds given unseen cDNA‐induced cell images. The mean and standard deviation values of Recall@1, Recall@3, Recall@5, and Recall@10 are reported. b) Cases on cDNA‐based graph retrieval task. Our model can identify diverse molecules that have similar functions to cDNA interventions (ticked).

One promising application of our model is the use of cellular images induced by non‐small molecule interventions (e.g. cDNA^[^ ^36^ ^]^) to identify small molecules with similar effects. This could compliment to traditional target‐based screening methods to find functional molecules at the cellular level. To mimic the phenotype‐based functional screening, we collect six sets of cellular images induced by cDNA interventions for functional genes from ref. [36] We use ExCAPEDB^[^ ^37^ ^]^ to retrieve functional (agonists) and non‐functional molecules of these six genes as the candidate pools. In particular, given a cell image induced by cDNA intervention, we use the pre‐trained model to rank the molecules in the corresponding candidate pool and evaluate the recall rate based on the top‐ranked functional molecules. As shown in **Figure** **4**a, our model effectively retrieves functional compounds given the unseen cellular images induced by cDNA interventions. Taking JUN as a reference (Figure 4), MIGA achieves a 0.24 ± 0.01 in recall@10, significantly outperforming random (0.08) and fingerprint (0.08 ± 0.01) baselines. This is consistent with our observation in the mutual retrieval tasks where the fingerprint‐based method cannot map the two distributions well. MIGA also has an obvious advantage over the best baseline CLIP (0.21 ± 0.01), indicating that the designed architecture can learn the representation correlations between molecules and images better. This trend holds for most gene comparisons. We observed two cases with relatively poor performance (recall@10 <0.10), which are mostly caused by the batch effect (e.g BRCA1), or low quality of images (HSPA5) produced by the respective genes, respectively. The results demonstrate that our model has the potentials to effectively bridge functional small molecules with other non‐small molecule therapeutics through the learned latent space, and thus have promising applications in drug repurposing or phenotype‐based virtual functional screening. More details can be found in the Section E (Supporting Information).

### 2.5 Benchmarking MIGA on Molecular Property Prediction Tasks

Another advantage of the cross‐modal pretraining model is the ability to transfer the learned information to downstream tasks. To evaluate the generalization ability of our model, we benchmark the performance on multiple challenging classification and regression tasks from the MoleculeNet. Herein, we compared our model with the SOTA SSL methods including EdgePred,^[^ ^38^ ^]^ ContextPred,^[^ ^10^ ^]^ AttrMasking,^[^ ^10^ ^]^ GraphCL,^[^ ^12^ ^]^ InfoGraph,^[^ ^39^ ^]^ GROVER,^[^ ^14^ ^]^ GraphLoG,^[^ ^11^ ^]^ GraphCL,^[^ ^12^ ^]^ and JOAO.^[^ ^13^ ^]^ Note that all the SSL models are re‐trained using released source code on CIL‐750K molecular data set. For fine‐tuning, we follow the same setting with refs. [12, 13] As summarized in **Table** **2**, MIGA performs the best on five out of six datasets and outperforms the existing SSL methods in the average performance by a large margin. The slightly lower performance in ESOL is expected as this property is related to the molecule's intrinsic characteristics, e.g., hydrophilicity and hydrophobicity. It does not rely on biological processes and phenotypic changes. Even though, MIGA will not cause negative transfer compared to non‐pretrained baseline. The performance validates that MIGA learns informative representations that can be transferred among different datasets.

**Table 2:** Comparison of SSL baselines against MIGA on six OGB datasets. Mean ROC‐AUC and Root Mean Squared Error (RMSE) (with the SD) of 5 times independent test are reported. The best and second best results are marked **bold** and **bold**, respectively.

Dataset	Classification (AUC)					Regression (RMSE)		
	HIV	Tox21	ToxCast	BBBP	Avg.	ESOL	Lipo	Avg.
Non‐pretrain	70.30 (0.51)	68.90 (0.80)	58.60 (1.20)	65.40(2.4)	65.80	1.278 (0.24)	0.744 (0.14)	1.011
ContextPred^[^ ^10^ ^]^	74.17 (1.33)	71.44 (0.11)	60.05 (0.15)	69.87 (0.99)	68.88	1.141 (0.03)	0.724 (0.02)	**0.933**
AttrMask^[^ ^10^ ^]^	75.55 (1.00)	74.58 (0.66)	59.51 (0.36)	68.88 (2.65)	69.63	1.194 (0.04)	0.736 (0.02)	0.965
EdgePred^[^ ^38^ ^]^	72.53 (1.20)	68.86 (0.38)	57.39 (0.80)	63.45 (1.39)	65.56	1.146 (0.05)	0.751 (0.02)	0.949
InfoGraph^[^ ^39^ ^]^	**76.22 (0.24)**	69.22 (0.78)	59.87 (0.35)	63.75 (1.52)	67.27	1.242 (0.01)	0.725 (0.01)	0.984
GraphLoG^[^ ^11^ ^]^	73.29 (2.64)	69.80 (0.41)	59.22 (1.05)	68.43 (2.86)	67.68	1.194 (0.02)	0.766 (0.01)	0.980
GraphCL^[^ ^12^ ^]^	74.85 (1.71)	74.19 (0.43)	61.37 (0.10)	66.13 (1.68)	69.14	1.151 (0.04)	0.745 (0.02)	0.948
GROVER^[^ ^14^ ^]^	74.35 (0.92)	74.02 (0.79)	61.30 (0.13)	**69.88 (0.58)**	**69.89**	1.199 (0.02)	**0.721 (0.01)**	0.960
JOAO^[^ ^13^ ^]^	74.91 (0.66)	**74.60** (0.49)	**61.62 (0.37)**	68.33 (0.58)	69.87	**1.117 (0.05)**	0.753 (0.02)	0.935
MIGA	**76.38 (0.55)**	**75.23 (0.71)**	**62.34 (0.23)**	**71.52 (0.43)**	**71.37**	**1.123 (0.01)**	**0.717 (0.00)**	**0.919**

### 2.6 Benchmarking MIGA on Clinical Trial Outcome Prediction Tasks

We further tested our method on the clinical trial outcome prediction that aims to predict the clinical trial outcome (success or failure) of drug molecules. This task is extremely challenging as it requires external knowledge of system biology and trial risk from the clinical record. To study the effect of the proposed cross‐modal self‐supervised learning model in capturing high‐level system biology information, we fine‐tune the pre‐trained model on three benchmark datasets. As summarized in **Table** **3**, MIGA achieved the best performance with PR‐AUC and AUC averagely 1.67% and 3.97% higher than the two best SSL baseline JOAO on the three clinical outcome prediction datasets, respectively. This is consistent with our hypothesis that phenotypic features can help predict tasks that involve complex biological processes. Note that HINT utilizes clinical information as features for supervised graph training, allowing it to learn more biological priors and outperforming previous SSL methods that only consider chemical information only. Our method, however, is superior to HINT, proving our statement that phenotypic features can help with predictions for those tasks involving intricate biological processes.

**Table 3:** Performance comparison of MIGA and several baselines for phase‐level‐outcome predictions on TOP dataset. We report the mean (and standard deviation) PR‐AUC and ROC‐AUC of five times. The best and second best results are marked **bold** and **bold**, respectively.

Task	Phase I		Phase II		Phase III	
Metrics	PR‐AUC	AUC	PR‐AUC	AUC	PR‐AUC	AUC
LR	0.634 (0.007)	0.487 (0.006)	0.509 (0.014)	0.534 (0.017)	0.675 (0.010)	0.528 (0.003)
XGBoost	0.646 (0.003)	0.508 (0.006)	0.481 (0.004)	0.516 (0.007)	0.712 (0.009)	0.597 (0.015)
HINT	0.683 (0.015)	0.516 (0.005)	0.537 (0.004)	0.584 (0.003)	0.689 (0.003)	0.621 (0.006)
ContextPred	0.693 (0.006)	0.541 (0.019)	0.544 (0.019)	0.586 (0.003)	0.710 (0.023)	0.554 (0.036)
GraphLoG	0.681 (0.016)	0.539 (0.016)	0.550 (0.043)	**0.593 (0.043)**	0.719 (0.024)	0.554 (0.024)
GROVER	0.711 (0.015)	0.559 (0.024)	0.521 (0.005)	0.574 (0.011)	0.713 (0.013)	0.575 (0.028)
GraphCL	0.721 (0.020)	0.578 (0.018)	0.543 (0.008)	0.588 (0.004)	**0.721 (0.009)**	**0.594 (0.007)**
JOAO	**0.721 (0.019)**	**0.586 (0.018)**	**0.546 (0.018)**	0.587 (0.000)	0.720 (0.000)	0.563 (0.006)
MIGA	**0.758 (0.010)**	**0.601 (0.031)**	**0.562 (0.010)**	**0.605 (0.022)**	**0.729 (0.008)**	**0.654 (0.016)**

## 3 Conclusion

In this study, we present a novel cross‐modal graph pre‐training framework, MIGA, incorporating phenotypic features of cellular images during pre‐training. This approach introduces generalizable biological priors to graph neural networks, enabling them to be transferred to downstream biologically relevant tasks. The contributions are twofold: first, we define a novel cross‐modal pre‐training task and construct a benchmark dataset for evaluation and further research. Second, we propose a graph‐image pre‐training framework that improves molecular SSL through three well‐designed contrastive learning modules. These modules are complementary and helpful in learning common low‐dimensional embeddings for graphs and images, facilitating efficient transfer to various downstream tasks, such as graph‐image retrieval, clinical outcome predictions, and molecular property predictions. Our experiments on these tasks demonstrate that MIGA outperforms state‐of‐the‐art machine learning‐based and SSL methods, highlighting the power of cross‐modal pre‐training strategy in the molecular graph representation area.

While cellular images are indeed valuable, they primarily provide a macro‐level view of cellular responses and do not directly reveal molecular structural information. Therefore, we aim to integrate the rich morphological information in stained cell images with the precise molecular structures by aligning cross‐modal molecular graphs and images.

Furthermore, we discovered that our model has inference capability for non‐molecular induced cell images, as demonstrated by cDNA‐based molecular retrieval experiments. This provides a new virtual screening strategy for phenotype‐based drug discovery, i.e., bridging small molecule therapeutics to other therapeutics modalities through cell images. Our work provides an initial step toward systematically using high‐content imaging for drug discovery.

There are still some challenges that need to be addressed, such as inherent data noise and batch effect.^[^ ^26^ ^]^ These could be resolved by designing specific encoders for cellular images, optimizing cross‐modal fusion mechanisms, and introducing heterogeneous cross‐modal data to improve the performance of graph pre‐training. Furthermore, given the low cost of high‐content imaging, large‐scale data generation is needed to bring these methods to their full potential. Joint initiatives, such as the recent JUMP‐CP consortium, which promises to release morphological profiling data for over 140 000 chemical and genetic perturbations,^[^ ^40^ ^]^ could further facilitate the scale and application of our approach.

## 4 Experimental Section

### Pre‐training Dataset

The experiments on the Cell Painting dataset CIL introduced by M.A. Bray et al.^[^ ^20^ ^]^ and M.A. Bray et al.^[^ ^27^ ^]^ were performed. The dataset originally consisted of 919 265 cellular images collected from 30 616 molecular interventions. Each image contained five color channels that captured the morphology of five cellular compartments: nucleus (DNA), Endoplasmic reticulum (ER), nucleolus/cytoplasmic RNA (RNA), F‐actin cytoskeleton (AGP), and Mitochondria (Mito). A molecular intervention was photographed from multiple views in an experimental well and the experiment was repeated several times, resulting in an average of 30 views for each molecule. To keep the data balanced, each molecule was restricted to a maximum of 30 images and removed the untreated reference images, which was in line with practical application scenarios, resulting in a cross‐modal graph‐image benchmark containing 750K views. This benchmark was referred as CIL‐750K. Detailed pre‐process procedure and analysis of the pre‐train dataset can be found in Section A (Supporting Information).

### Downstream Tasks

The pre‐trained model was evaluated in three downstream tasks: graph‐image retrieval, clinical outcome prediction, and molecular property prediction. Details of the implementations and fine‐tuning hyperparameters are in Section B (Supporting Information).

- Graph‐Image Retrieval contains two tasks: 1) graph as query and image as targets (Image retrieval); 2) image as query and graph as targets (graph retrieval/scaffold hopping). The goal of this task was to demonstrate whether the learned embeddings were able to preserve the inherent relations among corresponding graph‐image pairs. The CIL‐750K dataset were randomly split into a training set of 27.6K molecules corresponding to 680K images, and the remaining data were held for testing. The held‐out data consisted of 3K molecules and the corresponding 50K images. the retrieval task was formulated as a ranking problem, which was more robust and flexible compared to traditional classification problem, especially for new molecules in drug discovery. For each molecule corresponding to multiple images, it was determined whether it matches one of the images as the ground‐truth for retrieving. That is, all images corresponding to the same molecule were regarded as the ground truth of the molecule. In the inference phase, given a query molecular graph in the held‐out set, images were taken in the held‐out set as a candidate pool and rank candidate images according to the L2 distance between the image embeddings and the molecular embeddings, and vice versa. The ranking of the ground‐truth image/graph could then be used to calculate AUC, MRR (mean reciprocal rank), and Hit@1, 5, 10 (hit ratio with cut‐off values of 1, 5, and 10). Each experiment was repeated three times, and the result of mean and standard deviation on the test set were reported when MRR on the validation set was maximized.
- Clinical Trial Outcome Prediction aimed to predict the clinical trial outcome (success or failure) of drug molecules. This task was extremely challenging as it required external knowledge of system biology and trial risk from the clinical record. The Trial Outcome Prediction (TOP) benchmark constructed by T. Fu et al.^[^ ^41^ ^]^ was used for model evaluation. After dropping the combination mediation, the benchmark contained 944, 2865, and 1752 molecules for Phase I, II, and III tasks, respectively. The data splitting proposed by T. Fu et al.^[^ ^41^ ^]^ was followed and precision‐recall area under the curve (PR‐AUC) and area under the receiver operating characteristic curve (ROC‐AUC) was employed to measure the performance of all methods.
- Molecular Property Prediction MIGA was further evaluated on six molecular property datasets: HIV, Tox21, BBBP, ToxCast, ESOL, and Lipophilicity. These datasets were introduced by Z. Wu et al.,^[^ ^1^ ^]^ and further benchmarked by OGB community^[^ ^42^ ^]^ for low‐resource graph representation learning. Each data set contained thousands of molecular graphs as well as binary/scalar labels indicating the property of interest. The OGB^[^ ^42^ ^]^ setting was followed and the scaffold splitting strategy was adopted with a ratio for train/valid/test as 8:1:1 during fine‐tuning.

### Structure and Image Encoders—Structural Encoder

A compound structure could be represented as an attributed graph G=(V,E), where |V|=n denotes a set of *n* atoms (nodes) and |E|=m denotes a set of *m* bonds (edges). $X_{v}\in R^{n\times d_{n}}$ is represented for the node attributes with *d*_*n*_ as the feature dimension of node and $E_{uv}\in R^{m\times d_{e}}$ for the edge attributes with *d*_*e*_ as the feature dimension of edge. A graph neural network (GNN) *f* ^*g*^ learned to embed an attributed graph *G* into a feature vector *z*_*G*_. The Graph Isomorphism Network (GIN) was adopted from ref. [3], where the node and edge attributes were propagated at each iteration. Formally, the *l*‐th iteration of a GNN is:

(1) $$\begin{array}{c} h_{v}^{\left(l\right)}=g_{U}^{\left(l\right)}\left(h_{v}^{\left(l-1\right)},g_{AGG}^{\left(l\right)}\left\{\left(h_{v}^{\left(l-1\right)},h_{u}^{\left(l-1\right)},X_{uv}\right):u\in N\left(v\right)\right\}\right) \end{array}$$

where $h_{v}^{\left(l\right)}$ are the representation of node *v* at the *l*‐th layer, N(v) is the neighborhood set of node *v*, $h_{v}^{\left(0\right)}$ is initialized with *X*_*v*_ encoding its atom properties. $g_{AGG}^{\left(l\right)}$ stands for the aggregation function and $g_{U}^{\left(l\right)}$ stands for the update function. After *L* graph convolutions, *h* ^*L*^ had captured their *L*‐hop neighborhood information. Finally, a readout function was used to aggregate all node representations output by the *L*‐th GNN layer to obtain the entire molecule's representation *z*_*G*_:

(2) $$z_{G}=\sum_{v\in V}READOUT\left(h_{v}^{\left(L\right)}\right)$$

### Image Encoder

For the cellular image *I*, residual convolutional neural networks were used(ResNet‐34)^[^ ^43^ ^]^ as the basic feature extractor *f* ^*i*^ because it is lightweight, widely adopted,and has proven performance. DenseNet,^[^ ^44^ ^]^ EfficientNet,^[^ ^45^ ^]^ and the recently proposed Vision Transformer (ViT)^[^ ^46^ ^]^ were implemented as comparisons. Their original implementation with a small modification was strictly followed, namely adding an additional projection layer before the final output. These models were initialized with weights pre‐trained on ImageNet.^[^ ^47^ ^]^ Each input image *I* was encoded into a 1D feature vector *z*_*I*_ for further fusion.

### Pre‐Training Framework

MIGA was pre‐trained through three contrastive objectives: graph‐image contrastive (GIC) learning, masked graph modeling (MGM), and generative graph‐image matching (GGIM).

### Graph‐image Contrastive (GIC) Learning

Aimed to pull embeddings of the matched molecule‐image pairs together while pushing those of unmatched pairs apart by maximizing a lower bound on the mutual information (MI) between the molecular graph and cellular image for the positive pairs. It was achieve by minimizing a symmetric InfoNCE loss^[^ ^48^ ^]^ to maximize a lower bound on MI(*G*; *I*) and MI(*I*; *G*). Formally, the graph‐image contrastive loss is defined as:

(3) $$L_{GIC}=-\frac{1}{2}E_{p(G,I)}\left[\;log\frac{exp(s\left\langle z_{G},z_{I}\right\rangle /\tau )}{\sum_{k\neq i}^{K}exp\left(s\left\langle z_{G},z_{I}^{k}\right\rangle \right)}+log\frac{exp(s\left\langle z_{I},z_{G}\right\rangle /\tau )}{\sum_{k\neq i}^{K}exp\left(s\left\langle z_{I},z_{G}^{k}\right\rangle \right)}\right]$$

Here, the similarity function *s*〈*z*_*G*_, *z*_*I*_〉 = <*f*_*v*_(*z*_*G*_), *f*_*w*_(*z*_*I*_) >, includes cosine similarity and euclidean distance, where *f*_*v*_ and *f*_*w*_ are two linear projection layers that embed representations to a common space. τ is a temperature parameter, *K* is a set of negative image samples that not matched to *G*.

### Masked Graph Modeling (MGM)

To simultaneously leverage the intra‐molecular graph information and strengthen the interaction between molecule and image, both the image and the partial molecular graph were further utilized to predict the masked sub‐patterns. Following the masking strategies of ref. [10], the atom/bond attributes were randomly masked and constructed the context graphs for each molecular graph. The surrounding partial graph structures were used along with the corresponding image information to predict the masked attributed subgraphs and the corresponding attributes. The goal was to pre‐train molecular GNN *f* ^*g*^ that could not only learn the context information of atoms in similar structures but also capture domain knowledge by learning the regularities of the node/edge attributes distributed over graph structure. Herein, the masked molecular graph was defined as *G* ^*msk*^ and the observed (unmasked) graph as *G* ^*obs*^. Therefore, the training graph‐image pair (*G*, *I*) was transformed to (*G* ^*msk*^, *G* ^*obs*^, *I*) and the objective of MGM is:

(4) $$L_{MGM}=E_{(G^{msk},G^{obs},I)∼D}\left[\log P\left(G^{msk}|\left(G^{obs},I\right)\right)\right]$$

where (*G* ^*msk*^, *G* ^*obs*^, *I*) is a randomly sampled graph‐image pair from the training set *D*. Thus, the MGM training is equivalent to optimizing the following equation:

(5) $$L_{MGM}=-\frac{1}{\left|D\right|}\sum_{\left(g_{k}^{msk},g_{k}^{obs},I_{k}\right)}^{D}\log\left[p\left({\hat{g}}_{k}^{msk}|f_{m}\left(g_{k}^{obs},z_{I_{k}}\right)\right)\right]$$

where the ${\hat{g}}^{msk}$ is the prediction from the observed graph *g* ^*obs*^ and image embedding *z*_*I*_. The function *f*_*m*_ is the molecule attributes and context graphs prediction model. The MLM training was minimized by a cross‐entropy loss because *g* ^*msk*^ is a one‐hot vocabulary distribution where the ground‐truth attributes/subgraphs has a probability of 1.

### Generative Graph‐image Matching (GGIM)

Aimed to further distinguish the “hard” negative graph‐image pairs that share similar global semantics but differed in fine‐grained details. This was achieved by the combination of a graph‐image matching (GIM) loss and a generative matching (GM) loss.

Inspired by J. Li et al.,^[^ ^49^ ^]^ a cross‐modal encoder *f*_*c*_ was first utilized to fuse two unimodal representations and produce a joint representation of the graph‐image pair and append a multilayer perceptron followed by softmax to predict the matching probability of *p* ^*m*^ = *f*_*c*_(*z*_*G*_, *z*_*I*_). This can be formulated as:

(6) $$L_{GIM}=E_{(G,I)∼D}\left[\log P\left(y^{m}|\left(G,I\right)\right)\right]$$

where *y* ^*m*^ is the ground‐truth label representing whether the graph and the image are matched or not matched. The expected log‐likelihood function could be defined as:

(7) $$L_{GIM}=-\frac{1}{P+N}\sum_{k}^{P+N}\left[y_{k}^{m}\cdot \log\left(p_{k}^{m}\right)+\left(1-y_{k}^{m}\right)\cdot \log\left(1-p_{k}^{m}\right)\right]$$

where *P* denotes the number of positive pairs and *N* denotes the number of negative pairs. In practice, *P*: *N* = 1: 1. Contrastive similarity was used (Equation 3) to sample the in‐batch “hard” negative pairs, where the molecular embeddings that were more similar to the image had a higher chance of being sampled (and vice versa). These pairs were hard to distinguish because there might exist other images different from the ground‐truth that reflected the molecular perturbation equally well (or better) because of the batch effect.^[^ ^50^ ^]^

Motivated by recent success in generative contrastive learning,^[^ ^51^, ^52^ ^]^ variational auto‐encoders were further employed (VAE)^[^ ^53^ ^]^ as generative agents to reduce noise from experiments and enhance the cross‐modal interaction among the hard negative pairs. In particular, the generative agents were asked to recover the representation of one modality given the parallel representation from the other modality. Herein, cross‐modal generation was performed from two directions. This GM loss function can be formulated as:

(8) $$\begin{array}{c} L_{GM}=-\frac{\lambda_{kl}}{2}\left(D_{KL}\left(q_{\phi }\left(z_{I}|z_{G}\right)∥p\left(z_{I}\right)\right)+D_{KL}\left(q_{\phi }\left(z_{G}|z_{I}\right)∥p\left(z_{G}\right)\right)\right)\\ +\frac{1}{2}\left(E_{q_{\phi }\left(z_{I}|z_{G}\right)}\left[\log p_{\theta }\left(z_{G}|z_{I}\right)\right]+E_{q_{\phi }\left(z_{G}|z_{I}\right)}\left[\log p_{\theta }\left(z_{I}|z_{G}\right)\right]\right) \end{array}$$

where λ_*kl*_ is the hyperparameter balancing the reconstruction loss. *p*(*z*_*I*_) and *p*(*z*_*G*_) is the prior of image embedding and graph embedding, respectively, and *q*_ϕ_(*z*_*G*_∣*z*_*I*_), *q*_ϕ_(*z*_*I*_∣*z*_*G*_) is the corresponding posterior. Finally, the full contrastive objective of MIGA is:

(9) $$L=L_{GIC}+L_{MGM}+L_{GIM}+L_{GM}$$

### Implementation Details

Here, the implementation details for pre‐training and retrieval tasks are described.

### Pre‐training

The pre‐training model consisted of a Graph Isomorphism Network (GIN) from ref. [3] with five layers and 300 hidden dimensions and a residual convolutional neural network (ResNet‐34)^[^ ^43^ ^]^ with 63.5M parameters. The model was pre‐trained for 100 epochs using a batch size of 1024 on 8 NVIDIA 3090TI GPUs. The Adam optimizer with an initial learning rate of 3e‐4 and a weight decay of 0.02 was used. Images with resolution of 128×128 were taken. The margin γ was set to 4. Pre‐training on 750k graph‐image pairs for MIGA took 8 h, far less than 26 h for ContextPred and 48 h for GraphCL on 280k molecules of GEOM‐Drugs. The hyperparameters in the MIGA model, including the weights for contrastive learning modules, model architecture, and usage of cellular image, are shown in Section F (Supporting Information).

### Graph‐Image Retrieval

The CIL‐750K dataset were randomly split into a training set of 27.6K molecules corresponding to 680K images, and the remaining of the data was held for testing. The held‐out data consisted of 3K molecules and the corresponding 50K images. The retrieval task was formulated as a ranking problem. For each molecule corresponding to multiple images, it was determined whether it matched one of the images as the ground‐truth for retrieving. That is, all images corresponding to the same molecule were regarded as the ground truth of the molecule. In the inference phase, given a query molecular graph in the held‐out set, images were taken in the held‐out set as a candidate pool and rank candidate images according to the L2 distance between the image embeddings and the molecular embeddings, and vice versa. The negative sampling rate was set to positive: negative = 1:100. The GraphCL^[^ ^12^ ^]^ and cross‐modal pre‐learning methods, CLIP^[^ ^33^ ^]^ and ALIGN^[^ ^32^ ^]^ as baselines. The encoder part of these methods was changed to the same setting as MIGA, but the decoder, training part, and technical tricks was not changed. After pre‐training, the pre‐trained model was used to output embeddings of molecular graphs and cellular images, then the candidate pool was ranked based on their L2 similarity. Experiments were performed five times with different seeds. The average of MRR, AUC, Hit@1, Hit@5, and Hit@10 were reported.

### cDNA‐based Zero‐shot Graph Retrieval

The MIGA was used to prioritize the functional molecules from a compound pool. This task mimiced the real‐world virtual screening scenario using morphological features observed when overexpressing a specific gene by cDNA interventions. The cellular images that were overexpressed with cDNA open reading frames for 6 genes by M.H. Rohban et al.,^[^ ^36^ ^]^ were collected, including BRCA1, HIF1A, JUN, STAT3, TP53, and HSPA5. The ExCAPEDB database was used^[^ ^37^ ^]^ to retrieve gene‐specific agonists and inactive molecules that was observed in the training set. For each gene, a candidate pool was constructed with 20 agonists (positive samples) and 100 non‐functional molecules (negative samples), denoted as *P*(*I*, *G*_*A*_, *G*_*N*_). The statistics collected dataset has been detailed in Section 2 (Supporting Information). For each gene, given a randomly selected input image *I* with the resolution of 128×128, the model was asked to rank the *G*_*A*_ in front of the *G*_*N*_. The Recall@1, Recall@3, Recall@5, and Recall@10 were used as the metric to evaluate the model in such a zero‐shot graph retrieval task. The Recall@N means the proportion of positive samples in top@N to total positive samples.

### Related work—Related Work—Self‐supervised Learning on Graphs

Graph self‐supervised pre‐learning attempted to obtain supervision in unlabeled data to learn meaningful representations that could be further transferred to downstream tasks.^[^ ^10^, ^11^, ^12^, ^13^, ^14^, ^15^, ^16^, ^39^, ^52^, ^54^, ^55^, ^56^, ^57^, ^58^, ^59^, ^60^ ^]^ In general, these methods fall into two categories: contrastive‐based methods and generative‐based methods.^[^ ^61^ ^]^ The former aims to generate different views from the original graph and perform intra‐graph contrastive learning,^[^ ^10^, ^11^, ^12^, ^13^, ^15^ ^]^ while the latter ones are trained in a supervised manner to generate masked sub‐patterns or attributes at the inter‐graph level.^[^ ^10^, ^14^ ^]^ CLIP^[^ ^33^ ^]^ and ALIGN^[^ ^32^ ^]^ demonstrate that dual‐encoder models pretrained with contrastive objectives on millions of image–text pairs can learn strong image and text representations for cross‐modal alignment tasks and zero‐shot image classification. These approaches achieve remarkable performance on molecular graph representation tasks, but lack the ability to predict the complex properties involved in intricate biological processes. Different from the previous works, this method leverages pairwise data from molecular and cellular image to improve the biological perception of the learned molecular representation in a novel cross‐modal pre‐training framework.

### Related Work—Cross‐Modal Pre‐training

Pre‐training strategies for multi‐modal tasks had attracted massive attention, with most of these efforts targeting visual‐language representation learning. Most of them could be grouped into two categories. The first category was to use multi‐modal encoders to capture the interaction between image and text embeddings.^[^ ^62^, ^63^, ^64^, ^65^, ^66^, ^67^ ^]^ Approaches in this category achieved remarkable performance, but most of them required high‐quality images and pre‐trained object detectors. The other category focused on learning independent decoders for different modalities^[^ ^32^, ^33^, ^68^, ^69^ ^]^ For instance, CLIP^[^ ^33^ ^]^ learned pairwise relationships between language and images by performing pre‐training on a large amount of web data using independent encoders connected by contrastive losses. More recently, CLOOME^[^ ^70^ ^]^ employed a CLIP‐like loss to pre‐train the cellular image encoder with molecular fingerprints. Different from their focus on the image encoder, MIGA aimed at extracting information from cellular images to make enhanced molecular representations and designed two complementary cross‐modal contrastive losses for information interaction in an end‐to‐end manner.

## Conflict of Interest

C.L, J.Z. and W.L. work directly or indirectly for Galixir.

## Author Contributions

S.Z. and J.R. contributed equally to this work. S.Z. and Y.Y. conceived and supervised the project. S.Z., J.R., J.Z. and L.Z. contributed to the algorithm implementation. S.Z., L.Z contributed to the visualization and server implementation. S.Z., J.R, and Y.Y wrote the manuscript. S.Z., J.R., J.X., and Y.Y. discussed and performed the rebuttal experiments. All authors were involved in the discussions and proofreading.

## Supporting information

Supporting Information

## Data Availability

Demo, instructions, and codes for MIGA are available at https://github.com/prokia/MIGA.
