# Supplementary material for: Cross‐Modal Graph Contrastive Learning with Cellular Images
Source: Adv Sci (Weinh). 2024 Jun 21;11(32):2404845. doi: 10.1002/advs.202404845 (PMC11348220; doi:10.1002/advs.202404845)
Supplement: Supplementary file 1 — Supporting Information [file ADVS-11-2404845-s001.pdf]

ADVANCED SCIENCE
Open Access

Supporting Information

for *Adv. Sci.*, DOI 10.1002/advs.202404845

Cross-Modal Graph Contrastive Learning with Cellular Images

*Shuangjia Zheng**, *Jiahua Rao, Jixian Zhang, Lianyu Zhou, Jiancong Xie, Ethan Cohen, Wei Lu, Chengtao Li and Yuedong Yang**

Supporting Information for Cross-modal Graph Contrastive Learning with Cellular Images

Shuangjia Zheng [†] * *Jiahua Rao* [†] *Jixian Zhang* *Lianyu Zhou* *Jiancong Xie* *Ethan Cohen* *Wei Lu* *Chengtao Li* *Yuedong Yang**

[†] *These authors contributed equally: Shuangjia Zheng and Jiahua Rao.*

Global Institute of Future Technology, Shanghai Jiaotong University University

School of Computer Science and Engineering, Sun Yat-sen University

Galixir Technologies

Department of Computer Science, Xiamen University

IBENS, Ecole Normale Supérieure, PSL Research Institute

* *Corresponding author(s). E-mail(s): shuangjia.zheng@sjtu.edu.cn, yangyd25@mail.sysu.edu.cn*

Contents

A CIL-750K details **2**

B Implementation details and hyperparameters. **2**
- B.1 Generative Graph-image matching. 2
- B.2 Clinical Outcome Prediction 3
  - B.2.1 Dataset 3
  - B.2.2 Baselines 4
  - B.2.3 Fune-tuning hyperparameter 4
- B.3 Molecular property Prediction 5
  - B.3.1 Dataset 5
  - B.3.2 Featurization Extraction 5
  - B.3.3 Fune-tuning hyperparameter 5
- B.4 Dataset of cDNA-induced graph retrieval studies 6

C Case study for graph retrieval **6**

D Case study for image retrieval **7**

E Case study for zero-shot graph retrieval **8**

F Ablation study **8**
- F.1 Loss Modules 8
- F.2 Cellular Images 8
- F.3 Model Architecture 8

A CIL-750K details

The original CIL dataset includes 919,265 five-channel fields of view containing 30,616 test compounds. It also includes metadata files which record morphological features for each cell in each image, both at the single cell level and at the population average level (i.e. per well); a workflow for image analysis to generate morphological features is also provided. Quality control indicators are provided as metadata, indicating fields of view that are out of focus or contain highly fluorescent material or debris. Chemical annotations are also provided for the application of compound processing. Figure S1 shows the molecular data distribution and the number of view per molecule in CIL dataset.
In CIL, each molecular intervention is imaged from multiple views in an experimental well and the experiment was repeated several times, resulting in an average of 30 views for each molecule. In order to keep the data balanced, we restricted each molecule to a maximum of 30 images, resulting in a cross-modal graph-image benchmark containing 750K views. Each view has a resolution of 692×520 pixels and 5 channels. These images were imaged with the ImageXpress Micro XLS automated microscope at 20×magnification. We resize the images to 128×128 without any cropping to fit the CNN models' input format. Figure S2 shows examples of molecules and corresponding images from the CIL dataset and Figure S3 shows the multiple views of a random selected molecule.

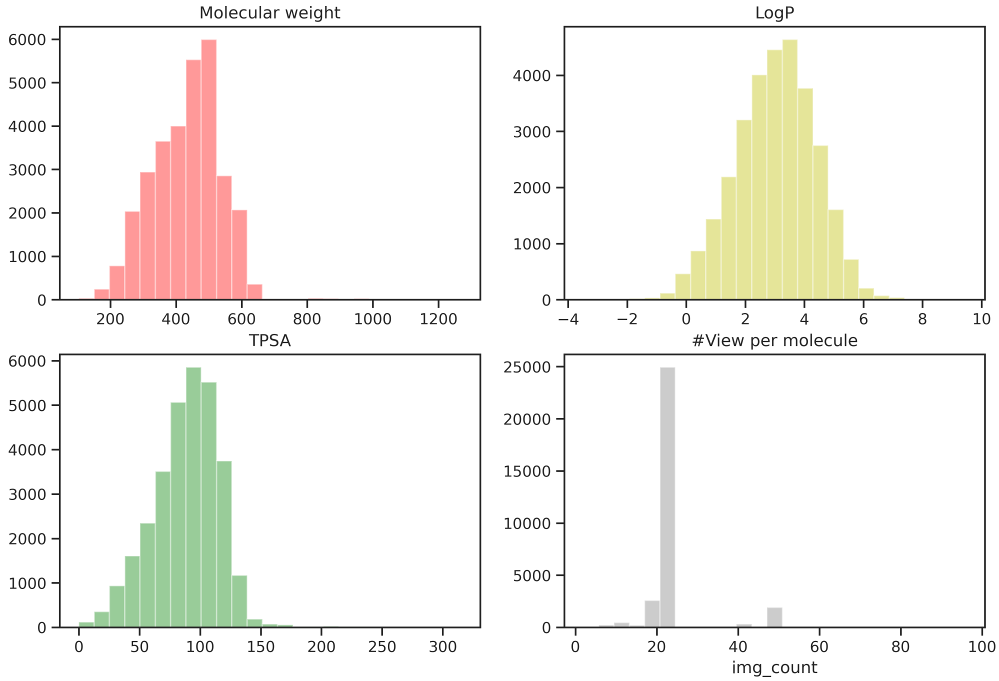

Figure S1: Data Distribution of CIL.

B Implementation details and hyperparameters.

Here we describe the implementation details for the pre-training and fine-tuning stages.

B.1 Generative Graph-image matching.

We employ Variational Auto-Encoders (VAE) as generative agents, which are asked to recover the representation of one modality given the parallel representation from the other modality. For example, we need to model the conditional likelihood $p(z_I \mid z_G)$ when generating the cellular image from their corresponding molecular graph. The reparameterized variable could be defined as $z_G = \mu_G + \sigma_G \cdot \zeta$ with mean μ_G, covariance σ_G, and $\zeta \sim \mathcal{N}(0, 1)$. Therefore, we have the following lower bound:

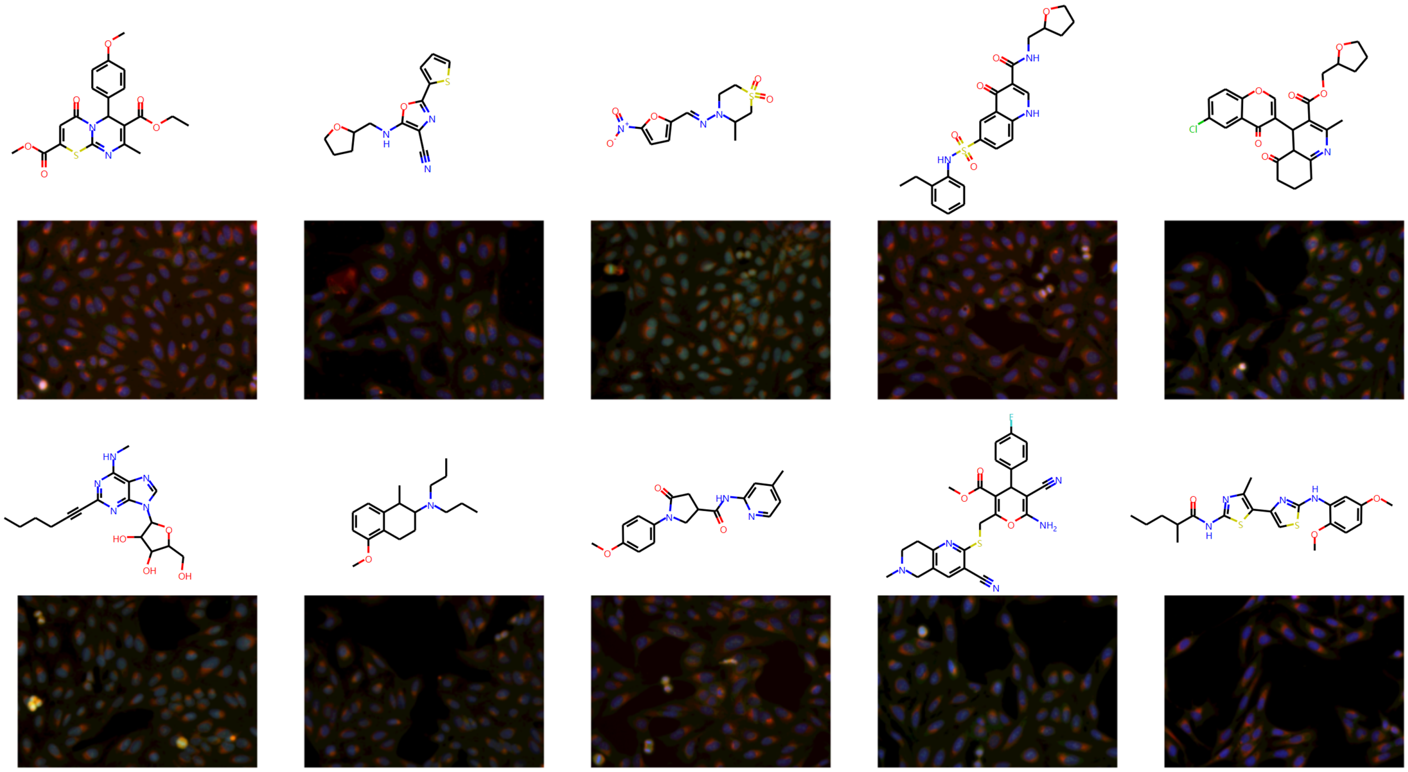

Figure S2: A random selection of 10 molecules and corresponding cellular images (1 view).

$$logp(z_I \mid z_G) \geq \mathbb{E}_{q(z_G|z_I)}[\log p(z_I \mid z_G)] - \mathcal{D}_{KL}(q(z_G \mid z_I) \| p(z_G)) \tag{1}$$

Similarly, when generating the molecular graph from their corresponding cellular image, we have:

$$logp(z_G \mid z_I) \geq \mathbb{E}_{q(z_I|z_G)}[\log p(z_G \mid z_I)] - \mathcal{D}_{KL}(q(z_I \mid z_G) \| p(z_I)) \tag{2}$$

Both the above objectives are composed of a conditional log-likelihood and a KL-divergence. Following the variation representation reconstruction (VRR) of [1], we use the mean-squared error (MSE) for reconstruction on the representation space:

$$\mathbb{E}_{q(z_G|z_I)}[\log p(z_I \mid z_G)] = \mathbb{E}_{q(z_G|z_I)}[\left\| z_G - q(z_G^{'} \mid z_I) \right\|_2^2] + C \tag{3}$$

$$\mathbb{E}_{q(z_I|z_G)}[\log p(z_G \mid z_I)] = \mathbb{E}_{q(z_I|z_G)}[\left\| z_I - q(z_I^{'} \mid z_G) \right\|_2^2] + C \tag{4}$$

Thus, combining both two regularizers mentioned above, the final GM loss function can be formulated as:

$$\begin{aligned} \mathcal{L}_{GM} = & -\frac{\lambda_{kl}}{2}(\mathcal{D}_{KL}(q_\phi(z_I \mid z_G) \| p(z_I)) + \mathcal{D}_{KL}(q_\phi(z_G \mid z_I) \| p(z_G))) \\ & + \frac{1}{2}(\mathbb{E}_{q(z_I|z_G)}[\left\| z_I - q(z_I^{'} \mid z_G) \right\|_2^2] + \mathbb{E}_{q(z_G|z_I)}[\left\| z_G - q(z_G^{'} \mid z_I) \right\|_2^2]) \end{aligned} \tag{5}$$

B.2 Clinical Outcome Prediction

B.2.1 Dataset

To standardize the clinical-trial-outcome predictions, we use the Trial Outcome Prediction (TOP) benchmark constructed by HINT, which incorporate rich data components including drug molecule information, disease information, trial eligibility criteria and trial outcome information. Herein, we consider

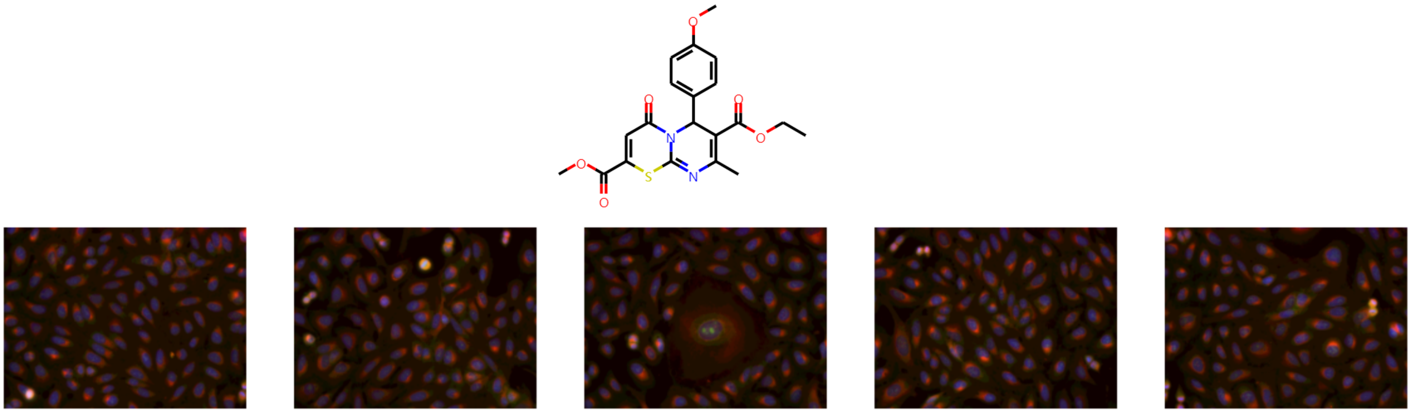

Figure S3: Five different views on the same molecule.

phase-level evaluation on the trial outcome, where we predict the outcome of a single-phase study. Since each phase has different goals (e.g., phase I is for safety, whereas phases II and III are for efficacy), we evaluate phases I, II, and III separately. We follow the data splitting proposed by HINT and data statistics are shown in Table S1.

Table S1: Statistics of Clinical Outcome Datasets.

Phase-level	Molecule	Successes	Failures
Phase I	944	564	380
Phase II	2865	1396	1469
Phase III	1752	1203	549

B.2.2 Baselines

We first include three machine learning-based methods (RF, LR, XGBoost) and a knowledge-aware GNN model HINT as our baseline. **Random Forest (RF)** is a bagging algorithm for classification or regression problems, which obtains the prediction by voting or averaging of each base learner (decision tree). **Logistic regression (LR)** is a simple, parallelizable classification method that uses maximum likelihood estimation for parameter estimation. **XGBoost**, also called an extreme gradient boosting tree, uses CART regression tree or linear classifier as a base learner to ensemble model predictions. These machine learning baselines utilize 1024-dimensional Morgan fingerprint features for trial outcome prediction. **HINT** is a hierarchical interaction network designed for clinical-trial-outcome predictions. It uses (1) 1024-dimensional Morgan fingerprint features, (2) a pre-trained BERT model to encode eligibility criteria into sentence embedding and (3) a graph-based attention model GRAM to encode disease information. Furthermore, we also include the self-supervised learning methods to constitute our baselines, including **ContextPred**, **GraphLoG**, **GROVER**, **GraphCL** and **JOAO**. For this downstream task, we use the molecule encoders over input molecule graphs for the fine-tuning of clinical outcome prediction.

B.2.3 Fune-tuning hyperparameter

For fine-tuning, an extra linear classifier is appended to the pre-trained GNN. We fine-tune the model for 100 epochs using a batch size of 32 with a dropout rate of 50%. We use the Adam optimizer with an initial learning rate of 1e-3. Experiments are performed for 5 times, with mean and standard deviation of ROC-AUC and PR-AUC are reported.

B.3 Molecular property Prediction

B.3.1 Dataset

BBBP: The Blood-brain barrier penetration dataset includes binary labels for 2035 compounds on their permeability properties. **Tox21**: The Tox21 dataset was created in the Tox21 data challenge, which contains qualitative toxicity measurements for 7821 compounds on 12 different targets, including nuclear receptors and stress response pathways. **HIV**: 41K compounds with binary labels for HIV virus replication inhibition. **ToxCast** includes 8576 drug compounds with binary labels of toxicity experiment outcomes with 617 targets. **ESOL**: The ESOL is a small dataset consisting of water solubility data for 1128 compounds. **Lipophilicity**: Experimental data for the octanol/water distribution coefficient of 4200 molecules.

Table S2: Statistics of datasets. GC for Graph Classification, GR for Graph Regression.

Dataset	Tasks	Type	Molecule	Metric
BBBP	1	GC	2,035	ROC-AUC
Tox21	12	GC	7,821	ROC-AUC
HIV	1	GC	41K	ROC-AUC
ToxCast	617	GC	8,576	ROC-AUC
ESOL	1	GR	1,128	RMSE
Lipophilicity	1	GR	4,198	RMSE

Table S3: Atom features

Features	Size	Description
Atom type	101	type of atom (e.g C,N,O)
Hybridization	6	sp, sp2, sp3, sp3d, sp3d2 or unknown
Number of H	1	number of bond hydrogen atoms
Degrees	1	number of neighbor atoms
Formal Charges	1	number of formal charge
Valences	1	number of valences

Table S4: Bond features

Features	Size	Description
Bond type	4	single, double, triple, aromatic
Stereo	6	none, any, E/Z or cis/trans
In ring	1	whether the bond is part of a ring
conjugated	1	whether the bond is conjugated

B.3.2 Featurization Extraction

The feature extraction contains three parts: 1) Node feature extraction. 2) Bond feature extraction. 3) Topology connection matrix. We use RDKit to extract all features as the input of GNN. Table S3 and Table S4 show the atom and bond features we used in MIGA.

B.3.3 Fune-tuning hyperparameter

For fine-tuning, we followed the GraphCL's [2] settings. An extra linear layer is appended to the pre-trained GNN to perform classification and regression, respectively. We fine-tune the model for 100 epochs using a batch size of 32 with a dropout rate of 50%. We use the Adam optimizer with an initial learning rate of 1e-3. Experiments are performed 5 times, with means and standard deviations of AUC and RMSE are reported.

B.4 Dataset of cDNA-induced graph retrieval studies

We collected cellular images that were overexpressed with cDNA open reading frames for 6 genes by [3], including BRCA1, HIF1A, JUN, STAT3, TP53 and HSPA5. We used ExCAPEDB database [4] to retrieve gene-specific agonists and non-functional molecules that have not been observed in the training set. (Table S5)

Table S5: Statistics of cDNA-induced graph retrieval datasets.

Gene	Functional cpds	Non-functional cpds	cDNA-induced Images
BRCA1	6937	9269	6
JUN	21	826	12
HIF1A	52	205	6
HSPA5	656	2498	12
TP53	3285	9364	12
STAT3	87	4179	18

C Case study for graph retrieval

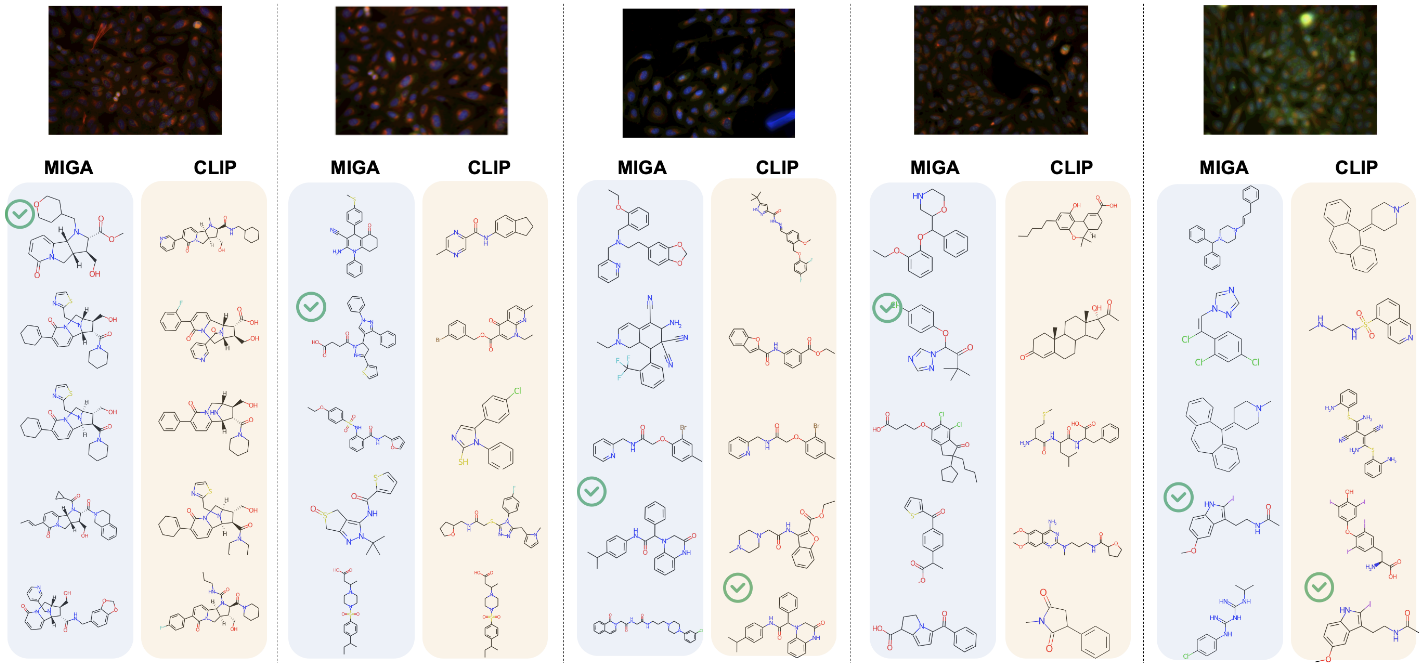

Figure S4: Additional examples for graph retrieval task. The top-ranked molecules retrieved by our method and baseline are shown. Molecules that hit the ground truth are flagged.

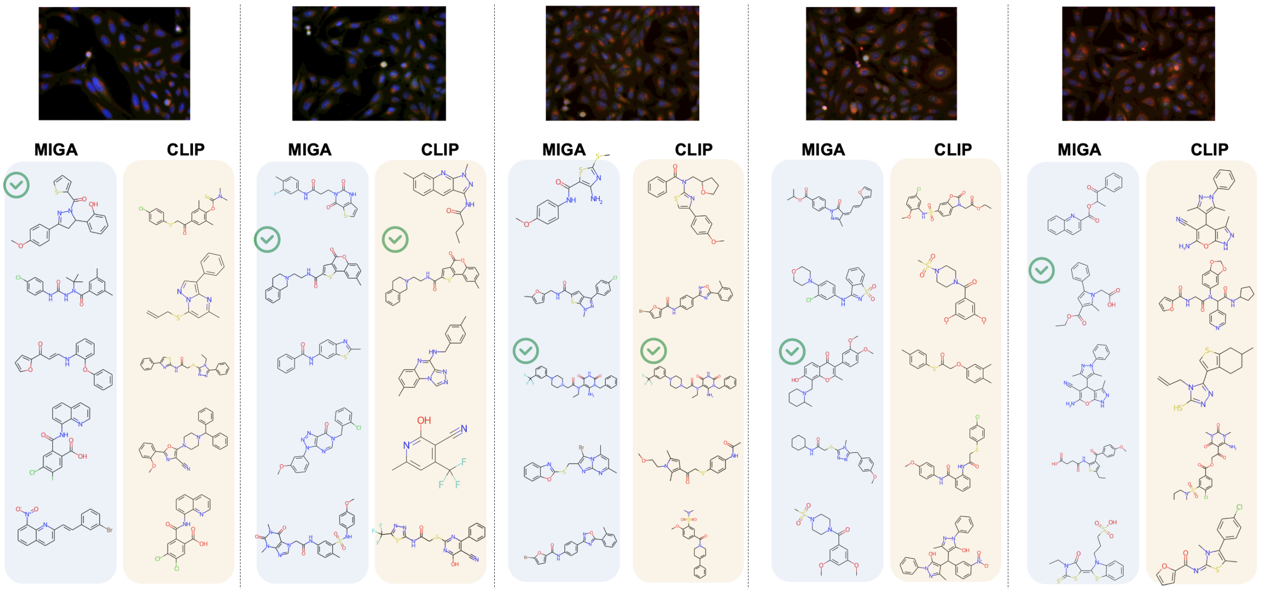

Figure S5: Additional examples for graph retrieval task. The top-ranked molecules retrieved by our method and baseline are shown. Molecules that hit the ground truth are flagged.

D Case study for image retrieval

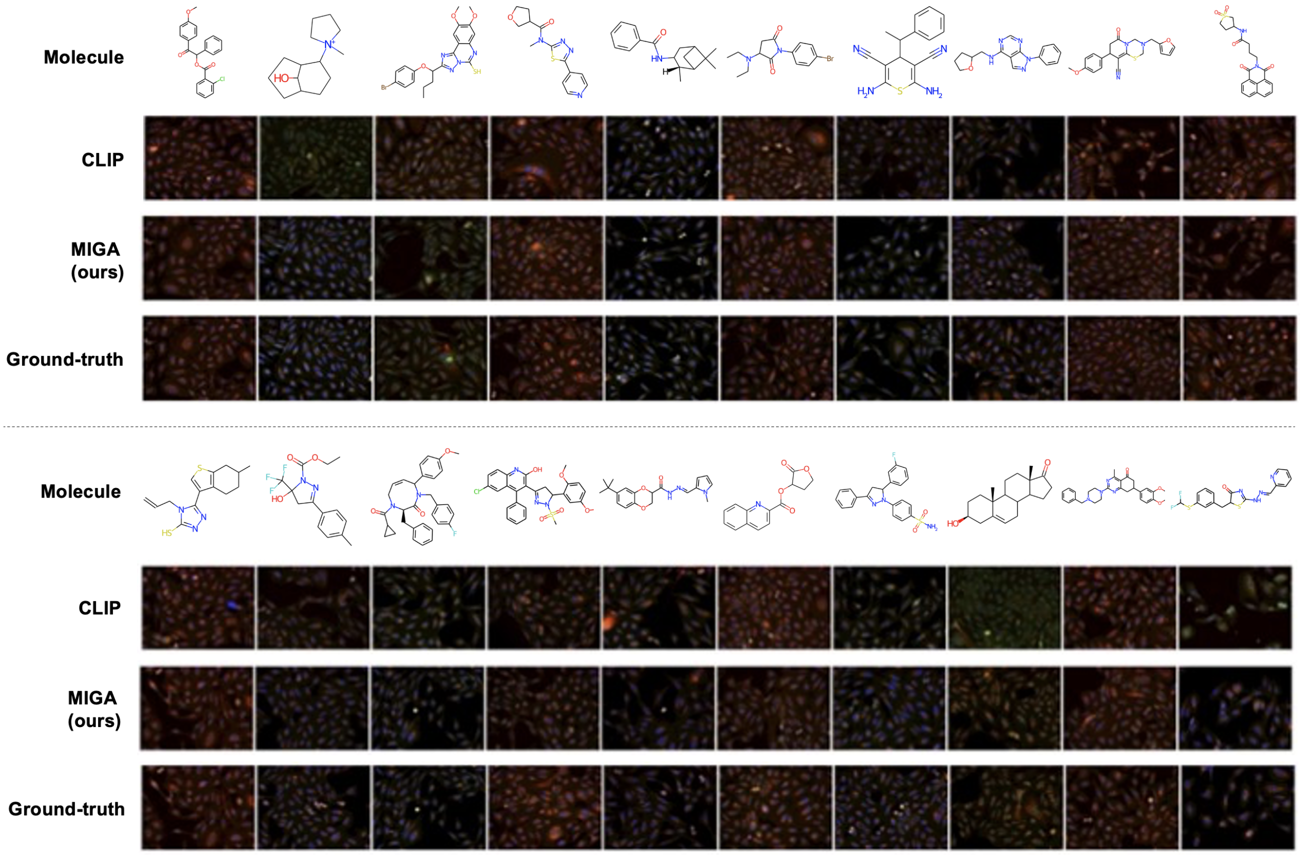

Figure S6: Additional examples for image retrieval task. The images retrieved by our method and baseline are shown.

E Case study for zero-shot graph retrieval

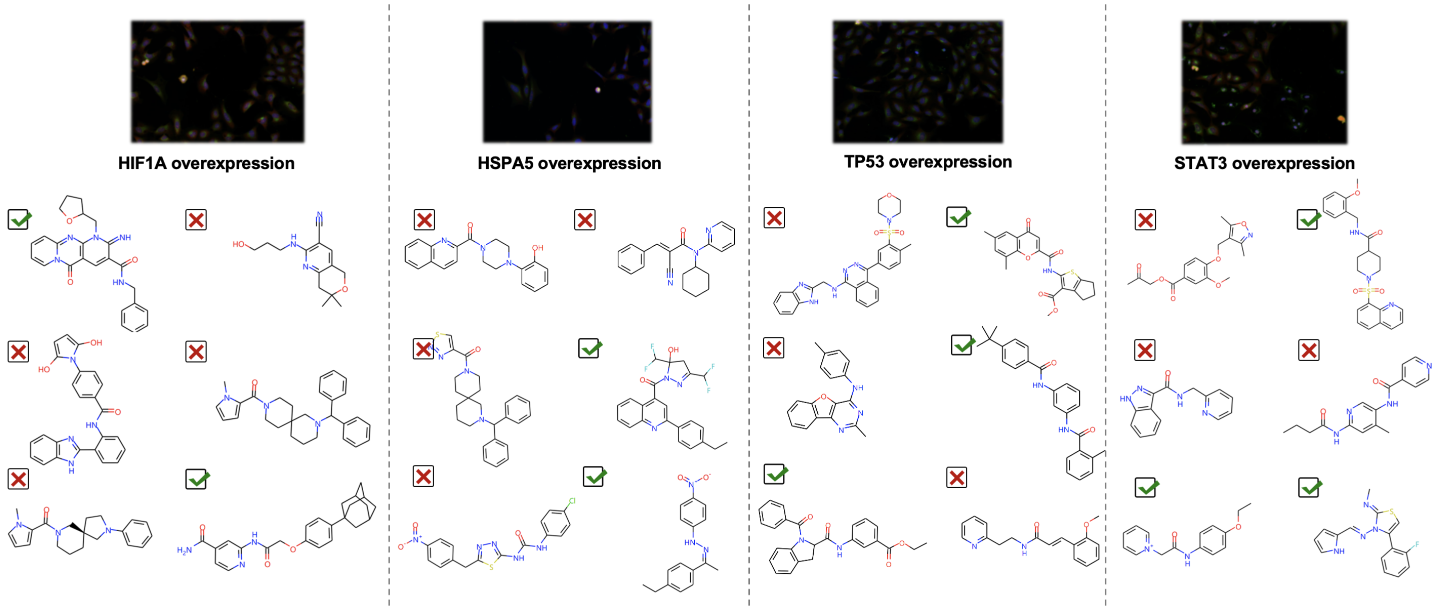

Figure S7: Additional examples for zero-shot graph retrieval task. The figure shows the cells induced by the cDNA interventions for specific genes (HIF1A, HSPA5, TP53, STAT3) and our model can identify diverse molecules that have similar functions to these cDNA interventions (ticked).

F Ablation study

F.1 Loss Modules

Table S6 shows the effect of the weights of each loss in our proposed framework, highlighting the importance of each component in the overall system. The results provides insight into the contribution of individual loss weights to the framework's efficacy. The variations in performance can be attributed to the interplay between the loss functions, which suggests that the synergy between different components is critical.
We also conducted the comparative experiments of the ablation variant GIC+GIM and CLIP pre-trained encoder on the molecular property prediction task (Table S7).

F.2 Cellular Images

Figure S8 (a) shows the effect of the number of view per molecule perform on graph retrieval task. We notice that with less than 10 views, the more views involve in pre-training the better the results would be, but using all views (on average 25 views per molecule) does not achieve more gains. We attribute this phenomenon to the batch effect of the cellular images, where images from different wells can vary considerably because of the experimental errors and thus mislead the model.

F.3 Model Architecture

Figure S8 (b) studies the impact of CNN architecture choices on the graph retrieval task. Due to the relatively small amount of data, we use small CNN variants (ResNet34[5], EfficientNet-B1[6], DenseNet121[7] and ViT_tiny[8]) for evaluation. We note that small CNN models such as ResNet, EfficientNet and DenseNet achieve comparable performance while bigger models like ViT do not show superiority. We assume this is because these models are pre-trained on non-cellular images, so heavier models do not necessarily lead to gains on cellular tasks.

Table S6: Ablation Study on Graph-image retrieval tasks.

Task / Metrics	Image Retrieval MRR	AUC	Hit@1	Hit@5	Hit@10
GIC	0.288	0.847	0.148	0.434	0.594
GIC+GIM	0.303	0.876	0.145	0.485	0.660
GIC+MGM	**0.409**	0.913	**0.244**	0.612	0.741
GIC+0.5*MGM+0.5*GGIM	0.383	0.919	0.222	0.618	0.729
0.5*GIC+0.5*MGM+0.5*GGIM	0.377	0.913	0.212	0.611	0.727
0.5*GIC+MGM+0.5*GIM	0.394	0.924	0.232	0.625	0.733
0.5*GIC+0.5*MGM+GGIM	0.399	**0.927**	0.242	**0.622**	0.742
MIGA (Full)	**0.417**	**0.936**	**0.248**	**0.623**	**0.748**

Table S7: Ablation Study on Molecular property tasks.

	Classification HIV	Tox21	ToxCast	BBBP
Non-Pretrain	70.30 (0.51)	68.90 (0.80)	58.60 (1.20)	65.40 (2.4)
CLIP	74.33 (0.87)	74.03 (0.35)	61.33 (0.18)	66.45 (1.82)
GIC+GIM	74.92 (0.46)	74.19 (0.24)	61.30 (0.16)	68.37 (0.74)
MIGA	**76.38 (0.55)**	**75.23 (0.71)**	**62.34 (0.23)**	**71.52 (0.43)**

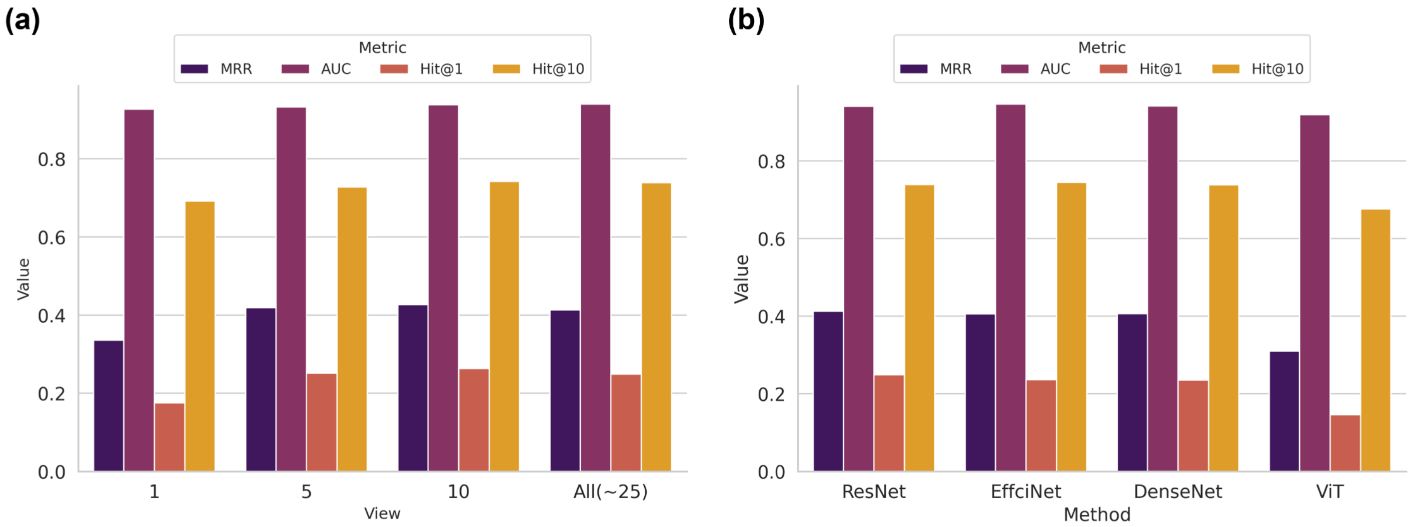

Figure S8: (a) Effect of number of view per molecule and (b) effect of CNN architecture.

References

[1] S. Liu, H. Wang, W. Liu, J. Lasenby, H. Guo, J. Tang, In *International Conference on Learning Representations*. **2022** .

[2] Y. You, T. Chen, Y. Sui, T. Chen, Z. Wang, Y. Shen, *Advances in neural information processing systems* **2020**, *33* 5812.

[3] M. H. Rohban, S. Singh, X. Wu, J. B. Berthet, M.-A. Bray, Y. Shrestha, X. Varelas, J. S. Boehm, A. E. Carpenter, *Elife* **2017**, *6* e24060.

[4] J. Sun, N. Jeliazkova, V. Chupakhin, J.-F. Golib-Dzib, O. Engkvist, L. Carlsson, J. Wegner, H. Ceulemans, I. Georgiev, V. Jeliazkov, et al., *Journal of cheminformatics* **2017**, *9*, 1 1.

[5] K. He, X. Zhang, S. Ren, J. Sun, In *Proceedings of the IEEE conference on computer vision and pattern recognition*. **2016** 770–778.

[6] M. Tan, Q. Le, In *International conference on machine learning*. PMLR, **2019** 6105–6114.

[7] G. Huang, Z. Liu, L. Van Der Maaten, K. Q. Weinberger, In *Proceedings of the IEEE conference on computer vision and pattern recognition*. **2017** 4700–4708.

[8] A. Dosovitskiy, L. Beyer, A. Kolesnikov, D. Weissenborn, X. Zhai, T. Unterthiner, M. Dehghani, M. Minderer, G. Heigold, S. Gelly, et al., *arXiv preprint arXiv:2010.11929* **2020**.
